# Developmental and Psychological Profiles, Risk Assessment, and Recidivism in Online Versus Contact Child Sexual Offending: A Systematic Review

**DOI:** 10.3390/bs16101946

**Published:** 2026-10-20

**Authors:** Giulio D’Urso, Alessandra Giuliani

**Affiliations:** 1 Department of Law, Economics and Human Sciences, Mediterranea University of Reggio Calabria, 89124 Reggio Calabria, Italy; 2 Department of Psychology, University of Bath, Bath BA2 7AY, UK; ag3152@bath.ac.uk

**Keywords:** child sexual abuse material, child sexual abuse, online sexual offending, risk factors, risk assessment, recidivism

## Abstract

**Background:** Online child sexual offending, particularly offences involving child sexual abuse material (CSAM), represents a growing area of concern. However, uncertainty remains regarding whether individuals who engage in online offences differ meaningfully from those who commit contact child sexual abuse (CSA) in their developmental histories, psychological characteristics, risk profiles, and treatment needs. **Methods:** A systematic search was conducted in Scopus, Web of Science, and PubMed in accordance with Preferred Reporting Items for Systematic Reviews and Meta-Analyses (PRISMA) guidelines. Studies examining developmental, psychological, criminological, risk assessment, recidivism, or treatment-related characteristics of individuals involved in online and/or contact child sexual offending were eligible. Twenty-nine studies were included and synthesised narratively by developmental and clinical domain. **Results:** Evidence indicated both overlap and meaningful differentiation between online and contact offending. Childhood adversity was reported across offence groups, although sexual victimisation histories tended to be less prevalent among online than contact offenders. Broad personality traits showed limited differentiation, whereas clinician-rated psychopathy and antisocial characteristics were generally more pronounced among contact offenders. Online and mixed offenders were more consistently characterised by atypical sexual interests and broader paraphilic or hypersexual patterns, although substantial within-group heterogeneity was evident. Some studies supported the relevance of CSAM-specific instruments and dynamic risk measures, but the available evidence did not establish their consistent superiority over static tools developed primarily for contact offenders. Recurrence findings from three treatment cohorts were synthesised narratively because differences in outcome definitions, ascertainment methods, denominators, and observation periods prevented meaningful pooling or direct comparison of CSAM-related and contact offending. Limited uncontrolled prospective evidence suggested reductions in offence-supportive cognitions and psychological distress, alongside substantial attrition and continued CSAM-related offending in some samples. **Conclusions:** Developmental adversity, atypical sexual interests, antisociality, offence history, and self-regulatory difficulties appear to combine differently across online, contact, and mixed offending pathways. Risk assessment findings should be interpreted in relation to the population, outcome, and setting in which each instrument was evaluated. Further validation is needed to establish whether CSAM-specific instruments and dynamic measures improve prediction beyond existing approaches. The evidence base remains constrained by heterogeneous definitions, predominantly male and forensic samples, variable follow up periods, and limited prospective treatment research. Larger longitudinal studies using harmonised definitions and validated outcome measures are required to clarify developmental pathways, transitions between online and contact offending, and treatment effectiveness.

## 1. Introduction

Sexual violence represents a systemic failure on multiple fronts. On the one hand, it reflects the failure of systems to adequately protect victims; on the other, it highlights the failure of systems to prevent perpetrators from engaging in deviant behaviour. Sexual violence against children represents a major public-health and safeguarding concern with potentially serious psychological and physical consequences for victims. Research has therefore examined developmental, psychological, and criminological factors associated with people who commit sexual offences, including differences related to victim characteristics and offence modality. Historical typologies have distinguished contact sexual offending against children from offences involving adults and from non-contact or technology-facilitated forms of offending, but contemporary evidence increasingly indicates substantial heterogeneity within these categories. Individuals who commit contact offences against children have been described as differing from people who commit sexual offences against adults in interpersonal, sexual interest, and offence-related characteristics, although such group-level patterns should not be interpreted as deterministic profiles (Marshall, 2004; Robertiello & Terry, 2007). A particularly important distinction concerns contact-only, online/CSAM-exclusive, and mixed offending. Online offending may include the use, possession, or distribution of child sexual abuse material (CSAM), as well as technology-facilitated grooming, whereas mixed offending involves both online and direct contact behaviours (Sheldon & Howitt, 2007). Existing reviews indicate that people who use CSAM are heterogeneous in developmental history, sexual interests, antisociality, and offending trajectories (Barroso et al., 2026). Accordingly, the present review treats offence modality as a useful descriptive and risk-relevant dimension rather than as evidence of discrete or mutually exclusive offender types.

The literature has highlighted that contact sexual offenders may present not only with antisocial characteristics, but also with pronounced cognitive distortions, extensive use of moral disengagement mechanisms, and significant deficits in empathy, as well as developmental histories characterised by severe emotional neglect (e.g., Grilli et al., 2024). Furthermore, Elliott et al. (2009) found that contact sexual offenders, on average, exhibited greater victim empathy distortions and more pronounced cognitive distortions, whereas online sexual offenders showed greater identification with fictional characters. The meta-analysis by Babchishin et al. (2015) further indicated that antisocial traits were more pronounced among contact sexual offenders than among online sexual offenders. However, the literature also points to a degree of homogeneity across these groups in terms of social cognitive and psychological deficits, suggesting that, despite important differences in the severity and nature of their characteristics, online and contact sexual offenders may share several underlying vulnerabilities.

In this sense, the Adverse Childhood Experiences (ACE) framework may provide a useful perspective for understanding sexual violence by exploring its possible developmental antecedents in early childhood experiences, including maltreatment and abuse, histories of institutionalisation, and highly emotionally and socially deprived environments (e.g., Chopin et al., 2023). From this perspective, adverse childhood experiences may contribute to the development of maladaptive interpersonal and emotional patterns that may subsequently interact with other individual and environmental risk factors (Petruccelli et al., 2022). In addition, the framework of cognitive distortions may help explain how socially acquired attitudes and beliefs can develop into maladaptive cognitive schemas that serve to justify violent behaviour and, consequently, legitimise it (D’Urso et al., 2018; D’Urso et al., 2019). The literature suggests that previous experiences of abuse, neglect, abandonment, and institutionalisation may represent risk factors in the development of deviant behaviour (Petruccelli et al., 2017; D’Urso et al., 2023). At the same time, factors associated with social learning, such as moral disengagement and cognitive distortions, may interfere with the development of adaptive socio-relational schemas. Rather than merely representing consequences of adverse childhood experiences, these mechanisms may amplify and consolidate deviant sexual schemas within the individual’s cognitive and emotional system (Petruccelli et al., 2022; Grilli et al., 2024).

Cognitive distortions may facilitate child sexual offending by enabling individuals to deny, justify, minimise, or otherwise neutralise harmful behaviour. Such offence-supportive beliefs are best understood as one component within a broader network of developmental, sexual, interpersonal, and self-regulatory factors rather than as a sufficient explanation for offending.

Developmental adversity is also relevant, although its role is heterogeneous. Childhood sexual victimisation has been associated with cognitive distortions and related psychological characteristics in some samples (Blank et al., 2018), while online-offending research has identified substantial childhood victimisation and poly-victimisation among some individuals who offend online (Chopin et al., 2022). More broadly, sexual offence samples may present with internalising distress, paraphilic interests, and other psychiatric difficulties (Jeglic et al., 2012; Booth & Gulati, 2014). These associations do not establish a causal pathway from childhood adversity or psychopathology to later offending, and neither factor is necessary or sufficient for sexual offending.

Taken together, these findings suggest that sexual violence should not be understood as the product of a single risk factor or developmental pathway. Rather, it may emerge from the complex interaction between adverse childhood experiences, social learning processes, cognitive distortions, psychopathological vulnerabilities, interpersonal difficulties, and contextual factors. Understanding how these dimensions interact in the development and maintenance of deviant sexual schemas may therefore be essential for improving risk assessment, prevention, and intervention strategies across both online and offline forms of sexual offending.

Considering these premises, and considering the ongoing controversies surrounding the assessment and treatment of sex offenders, it is important to address three research questions (RQs), which guide the present systematic review:

**RQ1:** What are the profiles and characteristics of individuals who commit sexual offences, and what risk factors characterise these populations? This question aims to identify potential differences between individuals who sexually abuse children online and those who commit contact (offline) sexual offences.

**RQ2:** What assessment tools have been used to evaluate these individuals, and what strengths and limitations do these instruments present?

**RQ3:** What treatment-oriented approaches have been studied among individuals who have engaged in, or are at risk of, online and/or contact child sexual offending, and what evidence is available regarding treatment engagement, treatment-related psychological changes, and subsequent recidivism? Within this framework, recidivism/recurrence was treated as a behavioural outcome relevant to RQ2 (risk assessment) and RQ3 (treatment-related follow-up), rather than as a separate research question. Throughout the review, we distinguish official reconviction or police record outcomes from confidential self-reported recurrence and behaviour observed during or after treatment, because these ascertainment methods capture non-equivalent outcomes.

For RQ3, studies reporting data relevant to treatment-related psychological changes and subsequent recidivism outcomes were considered. Consistent with the overall scope of the review, this research question was not intended to evaluate the efficacy of therapeutic interventions per se, but rather to examine the psychological and behavioural aspects relevant in the context of treatment. Therefore, it is included as part of the work, but this section is intended as a contextual, non-systematic synthesis.

## 2. Methods

### 2.1. Study Design and Search Strategy

This review was conducted in accordance with the PRISMA (Preferred Reporting Items for Systematic Reviews and Meta-Analyses) guidelines (Page et al., 2021) and followed the methodological guidance outlined in the Joanna Briggs Institute (JBI) Manual for Evidence Synthesis (Aromataris et al., 2024). The review protocol was prepared a priori but not prospectively registered. In line with JBI recommendations for systematic reviews and meta-analysis of aetiology and risk, we followed the following stages. The research questions were formulated using a Population, Exposure/Variables, Comparator, Outcome, Study design (PECOS) framework (Table 1), structured around three questions: (RQ1) what developmental, attachment, and personality factors are associated with online versus offline child sexual offending; (RQ2) how well do structured risk assessment tools perform in this population; and (RQ3) what is the evidence for treatment engagement, treatment-related psychological change, and treatment effectiveness (including recidivism during and after treatment) among online and offline offenders. PubMed, Scopus, and Web of Science were searched from inception to 24 July 2026, combining terms for (1) child sexual offending, (2) online/CSAM versus offline/contact offending, and (3) developmental, personality, and risk assessment constructs (full strings in Supplementary Materials).

PsycINFO was not searched as a separate database. Scopus and Web of Science were selected alongside PubMed to provide broad multidisciplinary coverage of psychological, criminological, and health literature. This choice should nevertheless be regarded as a limitation of search sensitivity, because database overlap does not guarantee retrieval of every PsycINFO-indexed record.

Three inclusion criteria were applied (see Supplementary Materials Table S1): (1) study design: peer-reviewed empirical studies using quantitative and/or qualitative methods, including cross-sectional, longitudinal/prospective, retrospective/archival, cohort, intervention, and instrument validation designs; (2) population: adolescents or adults identified via conviction, arrest, clinical/forensic referral, self-report of offending, or—where directly relevant to RQ1—community/non-forensic samples assessing sexual interest in children together with offence-related developmental or behavioural correlates; and (3) variables and outcomes: extractable findings concerning developmental, personality, cognitive, or psychological characteristics relevant to RQ1; the use, validation, predictive performance, or clinical application of risk assessment tools or procedures relevant to RQ2; and/or treatment engagement, attrition, psychological change, or subsequent offending relevant to RQ3. Treatment studies were eligible when they reported these RQ3 outcomes, even without a separate analysis of baseline developmental or personality characteristics. Comparative and single-group studies were eligible; a direct online-versus-contact comparison was not required. Population/community studies that did not provide a direct online-versus-contact comparison were retained only as contextual evidence and were identified as such in the synthesis. Victim-only studies without offending data, studies of adult-on-adult sexual offending without a child/CSAM component, general delinquency studies without relevant sexual-offending data, and case studies with fewer than five participants were excluded. Studies without extractable findings relevant to RQ1–RQ3, including those concerned solely with legal/sentencing outcomes, detection technology, or platform-level prevalence, were also excluded. Theoretical papers, editorials, commentaries, letters, and conference abstracts without full empirical reports were excluded; reviews and critical discussion were used for contextual discussion and citation searching rather than included in the primary synthesis. No restriction was placed on publication date. Terminology was retained at the level of the population actually studied. Thus, CSAM/CSEM users or offenders, online groomers, online-only offenders, contact offenders, and mixed/dual offenders are not treated as interchangeable categories; where studies used different operational definitions, those definitions are described, and findings are interpreted within that specific offence context.

### 2.2. Screening and Identification

**Figure 1:**
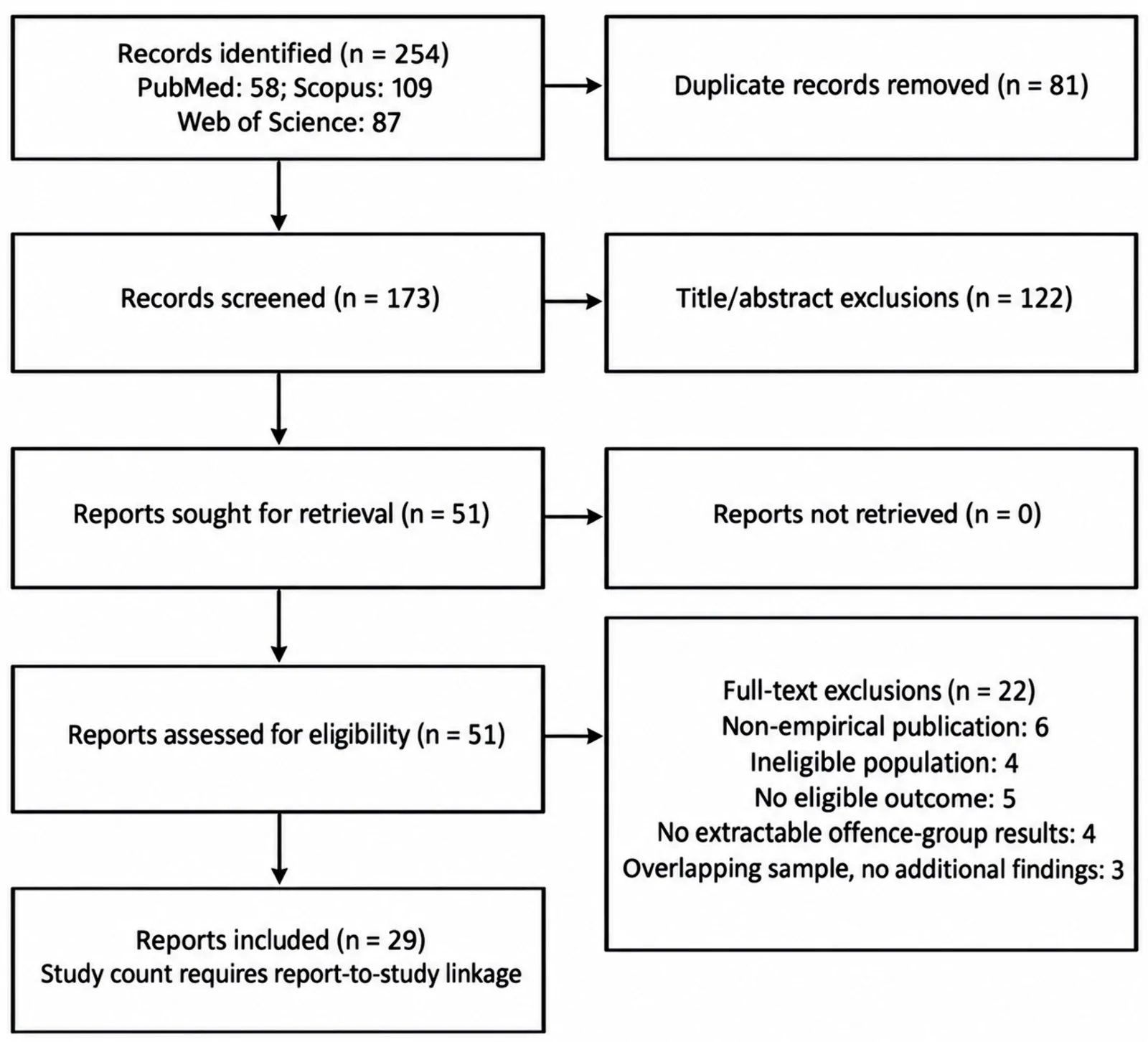
PRISMA flow diagram.

Searches identified 254 records: 58 from PubMed, 109 from Scopus, and 87 from Web of Science. After removing 81 duplicate records, 173 records remained for title-and-abstract screening. Titles and abstracts, and subsequently full texts, were screened independently against the prespecified eligibility criteria by two reviewers in Covidence (Veritas Health Innovation, Melbourne, Australia). Disagreements were resolved through discussion and consensus. A formal inter-rater agreement coefficient was not calculated. Of the 173 records, 122 were excluded, and 51 reports were sought for retrieval. All 51 reports were retrieved and assessed for eligibility; no reports were unavailable. Twenty-two reports were excluded: non-empirical publications (n = 6), ineligible populations (n = 4), no eligible outcomes (n = 5), no extractable offence-group results addressing the review questions (n = 4), and overlapping samples without additional eligible findings (n = 3). Twenty-nine reports were retained for extraction and synthesis (Figure 1).

### 2.3. Quality and Risk-of-Bias Assessment

Quantitative studies were appraised using the Quality in Prognosis Studies (QUIPS) tool (Hayden et al., 2013), considering study participation, attrition, measurement of prognostic factors and outcomes, confounding, and statistical analysis/reporting. Given the heterogeneity of the included quantitative evidence, which comprised different study designs and research objectives, QUIPS was used solely as a structured framework to guide the assessment of the main sources of methodological bias across studies. Its domains were applied pragmatically, considering the methodological characteristics and objectives of each study, rather than deriving a single uniform prognostic score. Instrument validation studies were additionally assessed with respect to sample representativeness, outcome base rates, criterion definition, and reporting of predictive accuracy indices. The only qualitative study (Wefers et al., 2024) was appraised using the CASP Qualitative Studies Checklist (Critical Appraisal Skills Programme, 2018). The appraisal was conducted independently by the two reviewers, with no disagreements arising during the consensus discussion. Quality assessment informed the interpretation of the findings rather than being used as a mechanical exclusion criterion. Study-level domain judgements are presented in Supplementary Table S2. These are provisional, design-informed judgements based on the study descriptions extracted for this review, not a newly verified independent full-text reassessment; they should be checked against each original report before being described as a repeated QUIPS assessment.

### 2.4. Data Extraction and Synthesis

A standardised extraction framework was used to record study design, setting, sample characteristics, offence group, developmental and psychological variables, risk assessment measures, treatment-related outcomes, follow-up duration, and reported effect estimates. Findings were first synthesised narratively and grouped thematically by research question rather than study-by-study. Quantitative pooling was undertaken only when at least two independent samples reported conceptually comparable effect estimates and sufficient information was available to estimate sampling variance. Odds ratios were pooled on the log scale, and proportions were pooled using the Freeman–Tukey double-arcsine transformation (Freeman & Tukey, 1950); DerSimonian-Laird random effects models (DerSimonian & Laird, 1986) were used because clinical and methodological heterogeneity was expected a priori. Statistical heterogeneity was assessed using Cochran’s Q and I^2^, and pooled estimates were back-transformed for reporting. Given the very small number of studies contributing to each meta-analysis, pooled estimates were treated as exploratory and interpreted primarily in relation to their confidence intervals, heterogeneity, and differences in outcome ascertainment. Where comparability was insufficient—for example, when instruments, outcomes, or follow-up windows differed materially—results were retained in the narrative synthesis.

After agreeing on the search strategy and inclusion criteria and removing duplicate records, the two authors independently conducted all subsequent stages of the review process, including full-text eligibility assessment, data extraction, and quality appraisal. The authors compared extracted information and resolved discrepancies through discussion and consensus. No disagreements arose at the article selection stage; therefore, calculation of inter-rater agreement was not considered necessary.

## 3. Results

### 3.1. Overview of Included Studies

Twenty-nine studies were included in the final synthesis. Twenty-eight used quantitative designs, and one used qualitative interviews (Wefers et al., 2024). Publication years ranged from 2007 to 2026. Sample sizes varied substantially, from 14 participants in the qualitative study to 8718 in the largest population-based survey (Dombert et al., 2016). Most offence comparison, risk assessment, and treatment studies comprised men recruited through forensic, correctional, probation, or specialist treatment settings; Fortunato et al. (2026) was the only female-only offender comparison (N = 254). A smaller set of studies recruited non-forensic men with sexual interest in children, anonymous Darknet CSAM users, or population/community samples. Two population studies (Alanko et al., 2013; Dombert et al., 2016) were retained as contextual evidence for RQ1 on developmental correlates, sexual interest, and self-reported offending, rather than as direct online-versus-offline comparisons. Study characteristics are summarised in Table 1.

Although adolescents were eligible under the prespecified population criterion, no included study provided an adolescent-only or adolescent-specific sample that met the review criteria. The included evidence therefore pertains overwhelmingly to adults. Female representation was similarly sparse: Fortunato et al. (2026) was the only dedicated female offender comparison. These gaps prevent meaningful developmental- or sex-specific comparison and identify adolescent and female populations as priorities for future research.

### 3.2. Quality Appraisal

Methodological limitations varied across studies (provisional study-level QUIPS judgements in Supplementary Table S2). The strongest evidence came from larger comparative forensic cohorts and longitudinal risk validation studies, but even these were constrained by low base rates for contact sexual recidivism and by the use of official records, which necessarily capture detected rather than all offending. RQ1 evidence was dominated by cross-sectional and retrospective designs, often using selected forensic or help-seeking samples and self-reported childhood adversity, sexual interests, or cognitions; these designs cannot establish temporal or causal developmental pathways. Several subgroup comparisons were modest in size, and definitions of online, CSEM/CSAM, contact, and mixed offending were not fully harmonised. Women were represented in only one dedicated study, and most evidence came from Europe or North America. Treatment studies were the most methodologically limited: cohorts were small, uncontrolled, and affected by attrition or completer selection. Accordingly, the synthesis below emphasises convergence across independent samples and distinguishes robust descriptive differences from exploratory or setting-specific findings.

### 3.3. Developmental, Personality, and Behavioural Characteristics

#### 3.3.1. Sociodemographic Characteristics

Across the direct forensic comparisons, online/CSEM offenders were generally younger than contact offenders. Webb et al. (2007) reported a mean age of approximately 38 years among internet offenders compared with 45 years among child molesters, while Jung et al. (2013) similarly described child pornography possessors as younger and more socioeconomically advantaged than contact offenders. These samples also suggested greater educational and vocational attainment among online offenders and a higher likelihood of being single or not living in a long-term cohabiting relationship. In Jung et al. (2013), child pornography possessors were more likely than comparison sexual offender groups to have attended post-secondary education and to be employed in skilled occupations, and they were less likely to have biological children. Webb et al. (2007) additionally found a higher proportion of White participants in the internet group than among contact child molesters. These findings are reasonably consistent with earlier descriptive literature, but they should not be treated as stable offender “profiles”: both studies are older, predominantly White male forensic cohorts and may partly capture cohort-specific access to technology, occupational opportunity, and detection practices rather than enduring developmental differences.

Population/community studies add context but do not provide direct online-versus-contact comparisons. Dombert et al. (2016), in an anonymous survey of 8718 German men, found that self-reported sexual fantasies involving prepubescent children were uncommon but not vanishingly rare, while behaviourally defined offending and a persistent paedophilic preference were substantially rarer. Alanko et al. (2013), using a population-based extended twin design in 3967 Finnish men, found evidence that sexual interest in youth under 16 showed modest familial/genetic influence. These studies broaden understanding of sexual interest development beyond detected offender samples, but neither should be interpreted as demonstrating an online offender subtype.

#### 3.3.2. Childhood Adversity and Developmental History

Evidence for a specific “abused–abuser” developmental pathway was inconsistent. Bailey et al. (2016), in 1102 minor-attracted men recruited online, found that childhood sexual abuse was associated with self-reported offending history, including combined online/contact offending. However, the study was cross-sectional and relied on retrospective self-report, so it cannot determine whether childhood sexual abuse causally increases later offending or whether the association reflects broader developmental adversity and correlated vulnerabilities. Boillat et al. (2017) provided a more differentiated account: in a small comparison of contact CSA offenders, CSEM users, and adult-preferring controls, childhood maltreatment was associated with neuroticism, and neuroticism rather than a particular abuse subtype appeared more relevant to offending group differences. Soldino et al. (2024), within contact offenders, found limited evidence that childhood abuse history distinguished those with minor versus adult victims. Together, these studies suggest that adversity is relevant for some individuals but is neither necessary nor sufficient to explain online or contact offending.

Fortunato et al. (2026) was the only dedicated female comparison. Among 254 females processed by police for online or offline child sexual abuse, a lifetime history of sexual victimisation was less common in the online group after adjustment (OR = 0.38, 95% CI [0.17, 0.86]).

Webb et al. (2007) and Fortunato et al. (2026) both provided direct online-versus-offline comparisons of sexual victimisation history, but the studies were not pooled because the exposures were not equivalent: Webb et al. examined childhood sexual abuse histories in male forensic groups, whereas Fortunato et al. examined lifetime sexual victimisation among female police-processed offenders. Considered narratively, both estimates were directionally consistent with less frequently reported sexual victimisation in online than contact/offline groups, but the evidence is too heterogeneous and limited to support a common pooled effect or a general developmental distinction between offence modalities.

#### 3.3.3. Personality, Psychopathy, and Interpersonal Functioning

Broad personality pathology showed substantially more overlap than separation between online and contact offenders. Jung et al. (2013) found that child pornography possessors, non-contact sexual offenders, and contact child molesters differed on only a small minority of broad personality indices. Kleijn et al. (2025) similarly found no clear CSEM-versus-contact separation across multiple domains of personality functioning, psychopathic traits, and narcissism; both offender groups were more clearly differentiated from community controls by difficulties in identity and relational functioning than from each other. Boillat et al. (2017) likewise found limited evidence for a distinct NEO-PI-R personality profile specific to CSEM use. However, in Webb et al. (2007), contact child molesters scored higher than internet offenders on clinician-rated psychopathy, whereas self-report personality pathology showed much less differentiation. This cross-study pattern suggests that apparent group differences may be stronger for externally rated antisociality and criminal lifestyle than for broad self-described personality traits. Overall, online-only groups tend, on average, to show lower antisocial/criminal history burden than contact or mixed groups while still displaying substantial within-group heterogeneity.

#### 3.3.4. Sexual Interests, Hypersexuality, and Offence-Supportive Cognitions

Sexual interest and self-regulation findings were more heterogeneous and appear to discriminate subgroups more clearly than broad personality traits. Kuhle et al. (2017), in 190 undetected pedo-/hebephilic men, found sexual preoccupation to be among the most robust dynamic correlates of child pornography and mixed offending. Neutze et al. (2011) likewise showed that sexual preference and dynamic risk factors jointly contributed to distinguishing undetected child pornography offending and contact sexual abuse, but no single dynamic factor produced a clean online/contact boundary. Konrad et al. (2018) found emotional congruence with children was not simply a marker of whether pedo-/hebephilic men had committed contact or non-contact offences, again suggesting that theoretically plausible risk constructs do not map neatly onto legal offence categories.

Recent evidence reinforces this heterogeneity. Lätth et al. (2026), among 160 anonymous Darknet CSAM users, reported high levels of hypersexuality and psychiatric comorbidity; hypersexuality was associated with greater time spent viewing CSAM, while some atypical sexual interests were associated with characteristics of the material viewed. Meißner et al. (2026), in a large forensic-evaluation sample, found that combined direct and indirect indicators of sexual interest differentiated offence subtypes better than any single component, with mixed offenders generally showing the strongest overall indications. Paquette and Cortoni (2021) found offence-supportive cognitions, atypical sexuality, and sexual coping/self-regulation were relevant across online and contact offending, but perceived anonymity and sexual preoccupation did not independently distinguish every group. Peschka et al. (2025) found childhood trauma was only weakly related to offence-supportive cognitions in voluntary treatment seekers.

Mental health difficulties also appear common. Lätth et al. (2026) reported substantial depressive, ADHD, autism-trait, hypersexual, and paraphilic symptom burdens in a self-selected Darknet sample, while Wild et al. (2020) described frequent psychiatric comorbidity among treatment-seeking men. Webb et al. (2007) found internet offenders were more likely than child molesters to have had adult mental health service contact.

#### 3.3.5. Behavioural Patterns and Typologies

Within detected IIOC/CSEM populations, offence history and access to children appear more informative than broad personality traits in distinguishing online-only from dual offenders. Long et al. (2013) and McManus et al. (2015) both found that characteristics of image collections, offending history, and access/opportunity were associated with contact offending. Dual offenders tended to have collections that were more closely linked to their contact offending, whereas non-contact offenders often had larger or more diffuse collections. These findings may have investigative value, but collection characteristics should not be treated as deterministic indicators of contact abuse because substantial overlap remained between groups.

A second typological framework distinguishes CSEM-exclusive, contact-only, and mixed/dual offenders. Across Babchishin et al. (2023), Elliott et al. (2019), Jung et al. (2013), and related studies, mixed offenders generally exhibited the highest levels of sexual interest indicators, criminal history, and recidivism risk, whereas CSEM-exclusive offenders showed lower levels of general antisociality and stronger offence specialisation. Fortunato et al. (2026) indicated that the pathways may differ among females: online offending was associated with being younger at the index offence and with co-perpetration, making male-derived typologies potentially incomplete.

Wefers et al. (2024) provided the only qualitative typology in the review. Interviews with 14 detected online groomers identified recurring themes of social connection, mental health difficulties, justification, secrecy, and technology, and three overlapping pathways centred on intimacy/social connection seeking, compulsive or addictive online behaviour, and fantasy or escape. Thirteen of 14 participants reported no intention of meeting the child in person. Although the sample is small and restricted to detected groomers rather than CSAM users broadly, these accounts add phenomenological depth to the quantitative distinction between online-specialised and mixed/contact pathways.

### 3.4. Risk Assessment and Reoffending

#### 3.4.1. Purpose-Built and Dynamic Risk Assessment

The most coherent risk assessment evidence concerned tools developed specifically for CSEM populations or measures of dynamic sexual offence risk. Eke et al. (2019) validated the Child Pornography Offender Risk Tool (CPORT) in an independent five-year sample of 80 men, in which 15% committed a new sexual offence and 9% a new child pornography offence. The CPORT showed moderate discrimination in validation and larger combined samples. Seto and Eke (2017) did not provide an independent validation of CPORT; rather, they developed and cross-validated the Correlates of Admission of Sexual Interest in Children (CASIC), a file-based proxy intended to identify evidence of pedo- or hebephilic interest when direct admission is unavailable.

Azizian et al. (2025) extended the construct validity evidence in 224 men on federal probation in California. CPORT scores converged moderately with a sexual recidivism measure (RM2000/S) while showing a much weaker association with a general criminality instrument (LS/CMI), supporting the intended sexual risk construct. However, because the study focused on convergent and divergent validity rather than prospective reoffending, it should not be cited as a predictive validity study. In a very different setting, von Franqué et al. (2023) found that among 132 voluntary treatment seekers with a history of CSAM, CPORT scores using CASIC modestly predicted future CSAM use (AUC = 0.63, 95% CI [0.53, 0.73]) and future contact CSA (AUC = 0.69, 95% CI [0.55, 0.82]). CPORT scoring based on clinical diagnosis was less consistent. This suggests that behaviourally anchored information may add value in confidential non-forensic samples, although discrimination remained modest.

Dynamic tools also performed at least as well in CSEM-exclusive groups as in contact offender groups. Babchishin et al. (2023), using a large prospective sample of 1350 adjudicated men, found that ACUTE-2007 and STABLE-2007 predicted recidivism among CSEM-exclusive offenders, with discrimination equal to or better than that observed in contact offenders. Webb et al. (2007) reported a similar direction for the earlier Stable-2000. These findings are clinically important because they suggest that changeable risk domains retain relevance in online-only cases, while also cautioning against relying exclusively on static contact offender instruments.

#### 3.4.2. Contact-Derived Static Tools and Limits of Transportability

Evidence for conventional static tools was less convincing when applied to CSEM-only or non-forensic samples. Elliott et al. (2019) found that offence history classification itself was strongly informative over approximately 13 years, but several treatment-relevant psychological domains and contact-derived risk indicators showed limited ability to distinguish subsequent reconviction among IIOC-only offenders. In von Franqué et al. (2023), STATIC-99 performed at chance level for future contact CSA within the subgroup with a CSA history (AUC = 0.49, 95% CI [0.29, 0.69]) and did not significantly predict future CSAM use. STATIC-C performed somewhat better for CSAM in the full sample but remained modest. Therefore, instruments calibrated on contact sexual offenders cannot be assumed to transport directly to confidential or CSEM-exclusive populations with different base rates and offence opportunities.

A formal meta-analysis of risk tool AUCs was not retained because the estimates across studies were not independent or outcome-equivalent: Seto and Eke (2017) validated CASIC against admission of sexual interest rather than recidivism, whereas CPORT, STATIC-99/RM2000/S, and dynamic tool studies used different outcomes and follow-up structures. Pooling these values would therefore create a deceptively precise summary of non-equivalent constructs.

#### 3.4.3. Reoffending Rates

**Official record** studies consistently suggest strong offence specialisation. Elliott et al. (2019) found that few IIOC-only offenders received subsequent contact/paraphilic reconvictions, while Jung et al. (2013) reported that child pornography recidivists reoffended with further child pornography offences. Similarly, in Babchishin et al. (2023), most sexual recidivism among CSEM-exclusive men remained CSEM-related. Thus, although escalation to contact offending can occur, detected online-only offenders show substantially lower contact-reoffending rates than mixed or contact offenders.

Recurrence was reported in three German non-forensic/treatment cohorts (Beier et al., 2024; von Franqué et al., 2023; Wild et al., 2020). However, the available extracted data do not establish comparable event numerators and at-risk denominators, outcome definitions, observation windows, and ascertainment procedures across studies. In particular, the denominator and assessment window underlying the Wild et al. recurrence percentages could not be verified against its small one-year follow-up sample (n = 9). Accordingly, these estimates are described narratively and are not pooled.

These differences also underscore the ‘dark figure’ of sexual offending: official conviction, reconviction, and police-contact data capture only detected and recorded behaviour and therefore cannot be assumed to represent the full incidence of sexual offending or victimisation. Confidential self-report may identify behaviour that are not documented in official records, but it is subject to different selection and reporting biases. Accordingly, **official record** recidivism and self-reported recurrence are treated as distinct outcome classes rather than interchangeable estimates of the same underlying rate.

Fortunato et al. (2026) qualify this predominantly male evidence: among females processed for online versus offline child sexual abuse, index offence type did not independently predict subsequent police contact. Offence specialisation therefore appears well supported in male samples, but cannot yet be assumed to generalise across sexes.

### 3.5. Treatment Engagement, Response, and Effectiveness

Treatment-related evidence was limited and methodologically heterogeneous. Five studies provided information relevant to treatment, support seeking, or outcomes among treated individuals (Beier et al., 2024; Elliott et al., 2019; von Franqué et al., 2023; Wefers et al., 2024; Wild et al., 2020), but only the German outpatient studies prospectively followed individuals accessing specialised voluntary services. These programmes were developed for people with a sexual interest in children who sought help to prevent CSA and/or CSAM. The principal intervention represented in this literature was the German Prevention Project Dunkelfeld and related specialised outpatient services. Treatment was multimodal and individually tailored, primarily combining cognitive–behavioural and sexological approaches targeting offence-supportive cognitions, sexual self-regulation, coping, and risk management strategies; pharmacological treatment could also be incorporated when clinically indicated. Accordingly, the available evidence concerns specialised multicomponent prevention programmes rather than a single standardised psychotherapy, and no included study compared treatment modalities experimentally.

Engagement emerged as an important limitation of the treatment evidence. Wild et al. (2020) reported substantial attrition across an outpatient treatment pathway. Of 122 individuals offered the diagnostic phase, 59 discontinued before completing treatment and a further 10 were excluded or expelled; only nine contributed complete pre-treatment, post-treatment, and one-year follow-up data. Thus, the longitudinal estimates were based on a small and highly selected subset of those initially presenting for treatment.

Long-term follow-up was similarly selective in Beier et al. (2024), who reassessed 56 individuals who had completed treatment within the Prevention Project Dunkelfeld, rather than the full population initially presenting to the service. von Franqué et al. (2023) likewise examined individuals accessing voluntary prevention services, with analyses restricted to those who progressed through the assessment and consent procedures. These studies demonstrate the feasibility of engaging individuals outside or before criminal justice detection, but also indicate substantial selection at referral, assessment, treatment completion, and follow-up. Consequently, subsequent outcome estimates principally characterise treatment-seeking individuals who remained engaged and cannot readily be generalised to all individuals at risk of CSAM or contact offending.

#### 3.5.1. Changes in Treatment-Relevant Psychological Domains

Wild et al. (2020) provided the clearest prospective evidence of psychological change during treatment. Offence-supportive cognitions, assessed using the Bumby Molest Scale, decreased significantly from pre- to post-treatment, with a large reduction in the larger pre–post sample and maintenance of improvement at one-year follow-up among the small number with complete longitudinal data. Overall psychological distress also decreased significantly, including after accounting for concurrent psychotherapy for comorbid difficulties. Subjective perceptions of being at risk of offending decreased in the larger pre–post sample.

Changes were not uniform across outcomes. General self-efficacy, perceived ability to control sexual impulses, and life satisfaction did not improve significantly, although relatively favourable baseline scores on some measures may have restricted the potential for detectable change. Overall, therefore, the available prospective evidence suggests that specialised outpatient treatment can modify some dynamic psychological targets, particularly offence-supportive cognitions, perceived risk, and psychological distress, but evidence for broader changes in functioning or sexual self-regulation is less consistent.

Importantly, these findings derive from uncontrolled pre–post comparisons. They cannot distinguish treatment effects from regression to the mean, repeated assessment, natural change, or selective retention of participants who responded favourably. Nor does improvement in an intermediate psychological target establish that such improvement reduces subsequent offending. Consistent with this distinction, Elliott et al. (2019), in a substantially larger UK community sample followed for approximately 13 years, found that treatment-relevant psychometric domains did not independently predict reconviction as strongly as offence history classification. Evidence that psychological targets are modifiable should therefore be distinguished from evidence that modifying those targets prevents reoffending.

#### 3.5.2. Recidivism During and After Treatment

The three treatment/non-forensic cohorts provide limited longitudinal behavioural evidence, but their recurrence estimates are not directly comparable. Observed contact offending during treatment or follow-up was consistently uncommon, whereas CSAM/CSEM recurrence remained substantially more frequent. However, these data cannot establish that treatment caused the low observed contact-offending rate because there were no randomised or matched untreated comparison groups, and participants were selected into help-seeking services. Similarly, high CSAM recurrence should not be interpreted as proof of treatment failure: confidential self-report detects behaviours that official reconviction studies miss, and follow-up lengths differed considerably.

Long-term follow-up nevertheless identifies an important limitation of current interventions. Beier et al. (2024) showed that CSAM use may persist or recur despite completion of a specialised prevention programme, whereas Wild et al. (2020) demonstrated improvement in several dynamic treatment targets without providing sufficient evidence to determine whether these changes translated into reduced offending. von Franqué et al. (2023), meanwhile, showed that offence-specific risk information retained prognostic value within voluntary treatment-seeking populations. Collectively, the evidence supports the feasibility of specialised intervention and suggests potential psychological benefits but does not yet establish efficacy in preventing either CSAM or contact sexual reoffending.

#### 3.5.3. Qualitative Evidence on Desistance and Support-Seeking

Wefers et al. (2024) complemented the quantitative treatment literature by describing participants’ own accounts of change. Positive offline relationships, disclosure to trusted others, reduced online anonymity, and access to non-judgemental support were described as protective. Participants also discussed a needs-based therapeutic approach as helping them recognise unmet social or emotional needs and pursue less harmful ways of meeting them. Importantly, most participants sought formal support only after police involvement rather than proactively. This finding aligns with the high attrition and self-selection seen in treatment cohorts and highlights access, stigma, confidentiality, and timing of help-seeking as central implementation issues rather than peripheral considerations.

## 4. Discussion

The present review provides an updated cross-domain synthesis of developmental and psychological characteristics, risk assessment, recurrence/recidivism, and treatment across online/CSAM and contact child sexual offending. It builds on prior reviews and comparative work by bringing these domains together within a common framework rather than claiming that the underlying areas have not previously been reviewed. The findings indicate partially differentiated patterns across offence modalities, but they do not support rigid or deterministic offender profiles. Rather, online-only, contact-only, and mixed offending appear to represent overlapping and heterogeneous behavioural pathways characterised by different average distributions of sexual interest, antisociality, offence history, and self-regulatory factors. The fact that the clinical profile is not always clearly defined and may be highly heterogeneous further increases the complexity of treatment. This means that each offender requires a thorough clinical and developmental assessment in order to identify and address the specific deficits and the broader psychopathological or symptomatic spectrum present. Such an individualised approach is essential to reduce the risk of recidivism and, above all, to prevent deviant impulses from prevailing when the individual is confronted with social situations and real-world interpersonal contexts.

In the older direct forensic comparisons, individuals who exclusively engaged in online/CSEM offending were generally younger, more educated, and less antisocial than contact sexual offenders. These differences may partly reflect cohort-specific access to technology, occupational opportunity, sampling, and detection practices, and should therefore not be interpreted as enduring characteristics of all people who offend online. Broad self-report measures of personality showed substantial overlap between groups, while clinician-rated psychopathy and criminal history indicators more consistently differentiated contact or mixed offenders from online-only groups. The evidence therefore supports probabilistic group differences rather than a categorical distinction between psychologically distinct online and offline populations.

Childhood adversity, mental health difficulties, atypical sexual interests, offence-supportive cognitions, and problems in self-regulation were reported across offence groups, but the largely cross-sectional and retrospective evidence cannot establish causal developmental pathways. Mixed online/contact offenders generally showed the greatest accumulation of sexual interest indicators and criminal history burden, whereas online-exclusive groups tended to show lower general antisociality and greater offence specialisation. These patterns are consistent with a dimensional and heterogeneous conceptualisation in which combinations of risk-relevant characteristics vary across individuals and offence modalities. Importantly, the reviewed literature did not consistently identify narcissistic or borderline traits as central distinguishing characteristics; their absence from group-level findings should not, however, be interpreted as evidence that they are irrelevant in every individual case.

Individuals who exclusively engage in CSEM offending also appear to show greater offence specialisation in **official record** studies. This pattern may reflect a more circumscribed detected offending trajectory, although the available evidence does not establish that online offending necessarily remains online or that escalation to contact offending cannot occur. Conversely, mixed offenders generally showed a greater accumulation of criminal history and sexual interest risk indicators. The relatively low observed rates of subsequent contact offending should therefore be interpreted as group-level findings that depend on sample selection, detection method, follow-up period, and offence definition rather than as evidence that contact offenders are inherently less likely to reoffend.

Regarding the second research question, the available evidence indicates that instruments developed specifically for CSEM offending and measures of dynamic risk may provide useful information in these populations, but the evidence does not establish their general superiority over traditional static instruments because comparable head-to-head validation studies are limited. The CPORT has demonstrated predictive and construct validity evidence across more than one sample, although its performance is moderate and context dependent. Similarly, the CASIC should primarily be interpreted as a behavioural indicator of the sexual interest component rather than as an independent actuarial risk assessment instrument.

ACUTE-2007 and STABLE-2007 also demonstrate satisfactory discriminatory capacity in samples composed exclusively of online sexual offenders. These findings support the potential value of dynamic risk assessment in this population, particularly when assessment is intended to identify potentially modifiable factors associated with recidivism. Nevertheless, self-report measures are subject to important limitations, including response bias, impression management, and social desirability. These concerns are particularly relevant in forensic contexts, where offenders may have strong incentives to minimise symptoms, deny problematic sexual interests, or present themselves in a socially desirable manner.

However, assessing the risk of recidivism among individuals convicted of sexual offences represents a critical component of forensic decision-making, treatment planning, and public safety management. Methodological research has increasingly demonstrated the superiority of structured risk assessment approaches over unstructured clinical judgement. In particular, the landmark meta-analysis by Hanson and Morton-Bourgon (2009), which examined more than 45,000 sexual offenders across 118 independent studies, provided compelling empirical evidence regarding the differential predictive accuracy of the main risk assessment approaches. The findings confirmed that empirically based actuarial instruments, such as the Static-99, demonstrate substantially greater predictive accuracy than unstructured clinical judgement, whose discriminative ability remains relatively modest across different types of offending, including sexual, violent, and general recidivism. Structured Professional Judgement (SPJ) approaches, such as the SVR-20, showed intermediate levels of predictive performance, offering a useful balance between methodological rigor and clinical flexibility. The meta-analytic evidence also highlights an important methodological limitation: the subjective clinical adjustment of actuarial scores (i.e., the adjusted actuarial approach) tends to reduce, rather than improve, the overall predictive validity of risk estimates. At the same time, the assessment of dynamic risk factors (e.g., including criminogenic needs, sexual deviance, and lifestyle instability) plays a crucial role. These factors not only contribute to the accuracy of medium- and long-term risk estimates but also represent key targets for treatment planning and rehabilitation.

Taken together, these findings support the prioritisation of objective, empirically validated assessment instruments in contemporary forensic practice, while assigning unstructured clinical opinion a strictly secondary role. In this regard, the study by Thornton et al. (2024) highlights the effectiveness of integrating dynamic protective factors into the assessment and treatment of men with a history of sexual offending. In contrast to traditional assessments that focus primarily on risk factors and deficits, the structured assessment of individual strengths offers several tangible benefits for recidivism prevention. The most relevant protective factors identified in the study converge around three main dimensions: Resilience, including self-control, stress management, and empathy; Adaptive Sexuality, encompassing self-regulation and prosocial sexual interests; and Prosocial Connections, including employment, stable relationships, and future-oriented goals. The study further demonstrates that the use of dedicated assessment tools, such as the Structured Assessment of Protective Factors against Sexual Offending (SAPROF), can support the personalisation of therapeutic pathways, in line with models such as the Good Lives Model. Moreover, incorporating protective factors can contribute to the development of less stigmatising case-management plans and provide a more comprehensive assessment of the extent to which risk has genuinely decreased following treatment.

Future research should evaluate whether multimethod assessment adds incremental value beyond structured risk instruments and file-based indicators. In particular, prospective studies could test whether carefully standardised clinical interviews, behavioural information, collateral records, and repeated measurement of dynamic risk factors improve prediction or case formulation in online/CSAM populations. Such approaches should be evaluated empirically rather than assumed to improve accuracy, particularly because assessment context and disclosure incentives differ markedly between forensic and confidential help-seeking settings.

The evidence on recurrence further highlights the importance of outcome ascertainment. The three non-forensic/treatment cohorts that incorporated confidential self-report reported substantially more CSAM-related recurrence than contact offending, whereas **official record** studies generally yielded lower detected rates. These patterns are consistent with the possibility that official records miss a proportion of subsequent offending, particularly offences that are difficult to detect, but the review cannot quantify the degree of under-ascertainment because the samples and follow-up procedures were not directly comparable.

Consequently, the substantial heterogeneity observed across studies prevents the identification of a single, universally applicable baseline recidivism rate. Recidivism estimates should therefore be interpreted in relation to the method of detection, the characteristics of the sample, the type of sexual offence, the length of follow-up, and the definition of recidivism adopted by each study.

Taken together, the risk factor literature supports considering offence modality, sexual interests, offence-supportive cognitions, self-regulation, antisociality, developmental adversity, and criminal history jointly rather than relying on a single defining characteristic. The present evidence does not establish a fixed sequence in which these domains should be addressed clinically. Instead, assessment and intervention should be individualised according to empirically supported risk and need factors, with particular attention to changeable domains where validated measures are available.

Regarding RQ3, the evidence concerning treatment remains preliminary. Limited uncontrolled prospective evidence suggests that offence-supportive cognitions, perceived risk, and psychological distress may improve over the course of specialist outpatient treatment, whereas evidence for broader changes in self-regulation is weaker. A meta-analysis of psychological treatment effects was not conducted because constructs, measures, samples, and follow-up periods were not sufficiently comparable. The exploratory meta-analysis reported in the Results instead pooled naturalistic recurrence proportions from three non-forensic/treatment cohorts; it should not be interpreted as an estimate of treatment efficacy. Across the treatment literature, the absence of randomised or matched untreated comparison groups, high attrition, selective follow-up, small longitudinal samples, heterogeneity in treatment exposure, and differing definitions of subsequent offending prevent causal conclusions about whether treatment reduces either CSAM-related or contact offending. The most defensible conclusion is therefore that specialised intervention is feasible and may modify some treatment-relevant psychological targets, while robust evidence of behavioural effectiveness remains insufficient.

The findings should be interpreted in light of several limitations. The evidence base was heterogeneous in design, assessment methods, terminology, sampling frame, outcome ascertainment, and follow-up duration. Most studies were cross-sectional or retrospective, limiting inference about developmental mechanisms, and most forensic and risk assessment samples consisted predominantly of men from Europe or North America. Population/community studies and confidential help-seeking samples were useful for contextualising detected offender cohorts but cannot be treated as equivalent populations. Findings derived primarily from male samples should not be generalised uncritically to women, for whom the available evidence remains sparse. Sex, offence modality, sampling frame, detection method, and cultural context should therefore be considered potential sources of heterogeneity and examined prospectively in larger, harmonised longitudinal studies. Moreover, the absence of adolescent-specific studies is particularly important because developmental stage may affect sexual behaviour, technology use, psychosocial functioning, risk formulation, and intervention needs; adult findings should therefore not be extrapolated to adolescents. Likewise, the single dedicated female study is insufficient to establish whether patterns derived largely from adult male samples generalise to women. Research designed specifically around adolescents and women is needed rather than treating these groups as extensions of the adult male evidence base.

In conclusion, although the study highlights a complex and heterogeneous clinical picture, it also underscores the need for a case-by-case clinical approach to assessment, aimed at minimising bias and identifying the most appropriate methods for evaluating recidivism risk. At the same time, greater attention should be devoted to the assessment of protective factors to prevent individuals from remaining trapped within distorted and psychopathological patterns of thinking and functioning.

## Figures and Tables

**Table 1:** Overview of included studies.

Study	Design/Sample/Groups	N	Country	Research Question
Alanko et al. (2013)	Population-based extended twin/family study examining childhood adversity and adult male sexual interest in youth under 16; contextual developmental evidence	3967	Finland	RQ1
Azizian et al. (2025)	Clinical field study examining convergent and divergent validity of the CPORT against RM2000/S and LS/CMI	224	USA	RQ2
Babchishin et al. (2023)	Prospective recidivism study; contact, CSEM-exclusive, and mixed groups; ACUTE-2007 and STABLE-2007	1350 (1042/228/80)	Canada	RQ2
Bailey et al. (2016)	Internet survey of minor-attracted men; correlates of self-reported offending history	1102	International	RQ1
Beier et al. (2024)	Prospective long-term follow-up of non-forensic treatment completers (1–11 years)	56	Germany	RQ1, RQ2, RQ3
Boillat et al. (2017)	Comparative study of contact CSA offenders, CSEM users, and adult-preferring controls; CTQ, NEO-PI-R, and MSI	66 (22/21/23)	Switzerland	RQ1
Dombert et al. (2016)	Population-based anonymous online survey of men examining self-reported sexual interest in and sexual behaviour involving prepubescent children; contextual prevalence evidence	8718	Germany	RQ1
Eher et al. (2019)	Incarcerated contact sexual offenders; child molesters versus rapists (contextual comparison)	1346	Austria	RQ1
Eke et al. (2019)	Five-year validation of the Child Pornography Offender Risk Tool (CPORT)	80; combined 346	Canada	RQ2
Elbert et al. (2022)	Forensic/archival study of federal sexual offenders; profile associated with CP possession/receipt	216	USA	RQ1
Elliott et al. (2019)	Long-term community follow-up; IIOC-only versus mixed IIOC/contact offenders; reoffending and risk factors	690 (584/106)	UK	RQ1, RQ2, RQ3
Fortunato et al. (2026)	Police record cohort comparing females processed for online versus offline child sexual abuse	254	Australia	RQ1, RQ2
Jung et al. (2013)	Comparative forensic study of CP possessors, non-contact sexual offenders, and contact child molesters	196 (50/45/101)	Canada	RQ1, RQ2
Kleijn et al. (2025)	Secondary analysis comparing CSEM, contact, violent, and community groups on personality functioning, psychopathy, and narcissism	270	Netherlands	RQ1
Konrad et al. (2018)	Community pedo-/hebephilic sample plus comparison group; emotional congruence with children	239 (217/22)	Germany	RQ1
Kuhle et al. (2017)	Community/non-forensic pedo-/hebephilic sample; sexual preference and dynamic risk factors	190	Germany	RQ1
Long et al. (2013)	Convicted IIOC offenders; dual versus non-contact offending and image collection characteristics	120 (60/60)	UK	RQ1
Lätth et al. (2026)	Anonymous Darknet-recruited CSAM users; psychiatric morbidity and offence-related behaviour	160	Sweden	RQ1
McManus et al. (2015)	Convicted IIOC offenders; factors associated with contact child sexual abuse	244	UK	RQ1
Meißner et al. (2026)	Forensic-evaluation sample; EISIP validity and sexual-interest patterns across offence subtypes	516	Austria	RQ1, RQ2
Neutze et al. (2011)	Community sample of pedophilic/hebephilic men; predictors of CP offending and contact CSA	155	Germany	RQ1
Paquette and Cortoni (2021)	Correctional sample; offence-supportive cognitions, atypical sexuality, self-regulation, and perceived anonymity	241	Canada	RQ1
Peschka et al. (2025)	Voluntary treatment-seeking sample; childhood trauma and offence-supportive cognitions	386	Germany	RQ1
Seto and Eke (2017)	Correlates of admitted sexual interest in children; development and cross-validation of the CASIC file-based proxy	286; cross-validation 60	Canada	RQ2
Soldino et al. (2024)	Incarcerated contact sexual offenders; comparison by adult versus minor victim age	97	Spain	RQ1
von Franqué et al. (2023)	Non-forensic treatment cohort; offence base rates and risk tools (STATIC-99/-C, CPORT/CASIC)	165	Germany	RQ1, RQ2, RQ3
Webb et al. (2007)	Comparative forensic assessment of internet offenders and child molesters; personality, psychopathy, and risk measures	210 (90/120)	UK	RQ1, RQ2
Wefers et al. (2024)	Qualitative interviews with detected online groomers; motivations, pathways, and support-seeking	14	UK	RQ1, RQ3
Wild et al. (2020)	Prospective outpatient treatment study with pre-/post-treatment and 1-year follow-up assessments	25; 1-year follow-up 9	Germany	RQ1, RQ2, RQ3

CSA = child sexual abuse; CSAM = child sexual abuse material; CSEM = child sexual exploitation material; CP = child pornography; IIOC = indecent images of children; CPORT = Child Pornography Offender Risk Tool; RM2000/S = Risk Matrix 2000/Sexual; LS/CMI = Level of Service/Case Management Inventory; CTQ = Childhood Trauma Questionnaire; NEO-PI-R = Revised NEO Personality Inventory; MSI = Multiphasic Sex Inventory; CASIC = Correlates of Admitted Sexual Interest in Children; EISIP = Explicit and Implicit Sexual Interest Profile. ACUTE-2007, STABLE-2007, STATIC-99, and STATIC-C are established names of sexual-offending risk assessment instruments and are therefore not expanded as acronyms.

## Data Availability

No new data were created or analyzed in this study. Data sharing is not applicable to this article.

## Supplementary Materials

The following supporting information can be downloaded at: https://www.mdpi.com/article/10.3390/bs16101946/s1, Table S1: Papers eligibility criteria (PECOS); Table S2: Study-level QUIPS domain judgements. L = low risk; M = moderate risk; H = high risk; NA = not applicable/insufficient information.
