# Supplementary material for: Developmental and Psychological Profiles, Risk Assessment, and Recidivism in Online Versus Contact Child Sexual Offending: A Systematic Review
Source: Behav Sci (Basel). 2026 Oct 20;16(10):1946. doi: 10.3390/bs16101946 (PMC13701439; doi:10.3390/bs16101946)
Supplement: Supplementary file 1 [file behavsci-16-01946-s001.zip › behavsci-4543123-supplementary.pdf]

Search run on 24/07/2026

PubMed

("child sexual abus*"[tiab] OR "child sex offend*"[tiab] OR "child molest*"[tiab] OR "sex offender*"[tiab] OR "child abuser*"[tiab] OR "Pedophilia"[Mesh])

AND

("online offend*"[tiab] OR "internet offend*"[tiab] OR "child sexual abuse material"[tiab] OR CSAM[tiab] OR "child pornography"[tiab] OR CSEM[tiab] OR "online grooming"[tiab] OR "contact sex offend*"[tiab] OR "contact sexual offen*"[tiab] OR "hands-on offender*"[tiab] OR "offline offend*"[tiab])

AND

("developmental trajector*"[tiab] OR "risk factor*"[tiab] OR "personality profile*"[tiab] OR "personality trait*"[tiab] OR "childhood adversity"[tiab] OR attachment[tiab] OR "risk assessment"[tiab] OR "Static-99"[tiab] OR recidivism[tiab] OR reoffending[tiab])

Scopus

TITLE-ABS-KEY ("child sexual abus*" OR "child sex offend*" OR "child molest*" OR "sex offender*" OR "child abuser*")

AND TITLE-ABS-KEY ("online offend*" OR "internet offend*" OR "child sexual abuse material" OR csam OR "child pornography" OR csem OR "online grooming" OR "contact sex offend*" OR "contact sexual offen*" OR "hands-on offender*" OR "offline offend*")

AND TITLE-ABS-KEY ("developmental trajector*" OR "risk factor*" OR "personality profile*" OR "personality trait*" OR "childhood adversity" OR attachment OR "risk assessment" OR "static-99" OR recidivism OR reoffending)

Web of Science

TS=("child sexual abus*" OR "child sex offend*" OR "child molest*" OR "sex offender*" OR "child abuser*")

AND TS=("online offend*" OR "internet offend*" OR "child sexual abuse material" OR CSAM OR "child pornography" OR CSEM OR "online grooming" OR "contact sex offend*" OR "contact sexual offen*" OR "hands-on offender*" OR "offline offend*")

AND TS=("developmental trajector*" OR "risk factor*" OR "personality profile*" OR "personality trait*" OR "childhood adversity" OR attachment OR "risk assessment" OR "static-99" OR recidivism OR reoffending)

Inclusion / Exclusion Criteria

Developmental Trajectories and Personality Profiles of Online vs. Offline Child Abusers

Population

Include: Adolescent or adult individuals who have sexually offended against children — contact/offline offenses, online offenses (CSAM production/possession/distribution, online solicitation/grooming), or mixed/crossover profiles. Identified via conviction, arrest, clinical/forensic referral, civil commitment, or self-report in community/help-seeking samples.

Exclude: Victim-only samples (risk factors for *being* abused, with no offending data on the same individuals); adult-on-adult sexual offending with no child/CSAM component; general delinquent youth samples with no sexual-offending element; case studies with N < 5.

Exposure / Variables of interest

Include (RQ1 – developmental/risk factors): Childhood maltreatment/adversity, attachment, family dysfunction, personality traits/disorders, cognitive distortions, empathy deficits, impulsivity, psychopathology — as predictors, correlates, or precipitants of offending onset, persistence, or recidivism.

Include (RQ2 – risk assessment): Use, validation, predictive accuracy, critique, or clinical application of risk assessment tools/procedures (e.g., Static-99R, STABLE-2007, SVR-20, RSVP, VRS-SO, CPORT, structured professional judgment).

Exclude: Studies with no extractable developmental, personality, or risk-assessment data (e.g., pure legal/sentencing-outcome studies; treatment-efficacy trials with no baseline offender characterization); studies focused only on detection technology or platform-level prevalence.

Comparator

Include: Online vs. offline vs. mixed offender comparisons; offender vs. non-offender comparisons; single-group descriptive studies (no comparator required, but tagged separately in extraction).

Exclude: Studies where the only comparison is by legal/offense-severity category with no developmental, personality, or risk-assessment content.

Outcomes

Any reported measure or finding relating to developmental history, personality, cognition, or risk-assessment performance (validity, accuracy, calibration, clinical utility) in this population.

Study design

Include: Peer-reviewed empirical studies — cross-sectional, longitudinal/prospective, retrospective/archival, cohort, and instrument-validation studies.

Exclude: Theoretical/conceptual papers with no original data; editorials, commentaries, letters, conference abstracts without full data; other systematic reviews/meta-analyses (retained separately for citation-chasing, not the primary synthesis).

Table S1. *Papers eligibility criteria (PECOS).*

**Domain**	**Criteria**
Population	Adolescents or adults who have sexually offended against children: contact/offline, online (CSAM production, possession, or distribution; solicitation/grooming), or mixed/crossover (identified via conviction, arrest, clinical/forensic referral, or self-report). For RQ1, community/non-forensic samples assessing sexual interest in children together with offence-related developmental or behavioural correlates were also eligible and retained as contextual evidence.
Exposure/Variables	(RQ1) Maltreatment, attachment, family dysfunction, personality, cognitive distortions, empathy, impulsivity, psychopathology, as correlates of offending onset, persistence, or recidivism. (RQ2) Validation, predictive accuracy, or clinical application of structured risk-assessment tools (e.g., Static-99R, STABLE-2007, SVR-20, RSVP, VRS-SO, CPORT). (RQ3) Participation in structured or self-referred treatment/intervention programmes, and pre–post or longitudinal change in treatment-relevant cognitions, distress, self-efficacy, or recidivism.
Comparator	Online vs offline vs mixed/crossover; offender vs non-offender; pre- vs post-treatment or vs follow-up within the same sample; single-group descriptive studies eligible and tagged separately. A direct online-versus-contact comparison was not required. Population/community studies were identified as contextual evidence for RQ1.
Outcomes	Any developmental, personality, cognitive, or risk-assessment (validity/accuracy/calibration/utility) finding within this population; and, for RQ3, treatment engagement/attrition, change in treatment-target psychological variables, and recidivism during or after treatment.
Study design	Peer-reviewed empirical studies: cross-sectional, longitudinal, retrospective/archival, cohort, qualitative, and instrument-validation designs.
Exclusions	Victim-only studies without offending data; adult-on-adult sexual offending without a child/CSAM component; general delinquency studies without relevant sexual-offending data; case studies with N < 5; studies without extractable findings relevant to RQ1–RQ3; theoretical/conceptual papers, editorials, commentaries, letters and conference abstracts without full empirical reports. Reviews and meta-analyses were retained for contextual discussion and citation searching rather than the primary synthesis.

Table S2. Study-level QUIPS domain judgements. L = low risk; M = moderate risk; H = high risk; NA = not applicable/insufficient information.

**Study**	**Participation**	**Attrition**	**Prognostic-Factor Measurement**	**Outcome Measurement**	**Confounding**	**Analysis**
Alanko et al. (2013)	M	NA	M	M	H	M
Azizian et al. (2025)	M	NA	M	M	H	M
Babchishin et al. (2023)	M	M	M	M	M	M
Bailey et al. (2016)	H	NA	M	M	H	M
Beier et al. (2024)	H	H	M	M	H	M
Boillat et al. (2017)	M	NA	M	M	H	M
Dombert et al. (2016)	H	NA	M	M	H	M
Eher et al. (2019)	M	NA	M	M	H	M
Eke et al. (2019)	M	NA	M	M	H	M
Elbert et al. (2022)	M	NA	M	M	H	M
Elliott et al. (2019)	H	M	M	M	H	M
Fortunato et al. (2026)	M	NA	M	M	H	M
Jung et al. (2013)	M	NA	M	M	H	M
Kleijn et al. (2025)	H	NA	M	M	H	M
Konrad et al. (2018)	H	M	M	M	H	M
Kuhle et al. (2017)	H	NA	M	M	H	M
Long et al. (2013)	M	NA	M	M	H	M
Lätth et al. (2026)	H	NA	M	M	H	M
McManus et al. (2015)	M	NA	M	M	H	M
Meißner et al. (2026)	M	NA	M	M	H	M
Neutze et al. (2011)	H	NA	M	M	H	M
Paquette & Cortoni (2021)	M	NA	M	M	H	M
Peschka et al. (2025)	H	NA	M	M	H	M
Seto & Eke (2017)	M	NA	H	M	H	M
Soldino et al. (2024)	M	NA	M	M	H	M
von Franqué et al. (2023)	H	NA	M	H	H	M
Webb et al. (2007)	M	NA	M	M	H	M
Wild et al. (2020)	M	H	M	H	M	H
